# Hypervigilance as a Specific Longitudinal Predictor of Anxiety Symptoms: A Self-Report Study

**DOI:** 10.1192/j.eurpsy.2026.11225

**Published:** 2026-07-31

**Authors:** C. Sorroche, L. Prat-Torres, P. Chavarría-Elizondo, V. De la Peña-Arteaga, I. Martínez-Zalacaín, A. Juaneda-Seguí, E. Vilajosana, J. Raduà, C. Soriano-Mas, M. À. Fullana

**Affiliations:** 1 Hospital Clínic de Barcelona; 2 Private Practice, Barcelona; 3 Institut d’Investigació Biomédica de Bellvitge, Hospitalet de LLobregat; 4 Institut de Recerca Sant Pau; 5 August Pi i Sunyer Biomedical Research Institute; 6 University of Barcelona, Barcelona, Spain

## Abstract

**Introduction:**

Hypervigilance is a distinctive attentional control state involving increased scanning of the environment to detect potential threats (Richards et al., Neurosci Biobehav Rev 2014; 47:102–117). Although it has been conceptually linked to anxiety, its specificity in predicting anxiety versus depressive symptoms remains unclear.

**Objectives:**

This study aimed to examine the discriminant predictive validity of hypervigilance over time, assessing whether it serves as a specific longitudinal marker for anxiety symptoms compared to depressive symptoms.

**Methods:**

A sample of 165 adults with varying levels of anxiety risk completed the Brief Hypervigilance Scale (BHS; Bernstein et al., Psychol Trauma 2015; 7:448–455) at baseline. Anxiety and depression symptoms were assessed six months later using the respective subscales of the Spanish version of the Depression Anxiety Stress Scales (DASS-21; Daza et al., J Psychopathol Behav Assess 2002; 24:195–205). Hierarchical regression analyses were conducted, controlling for age and baseline symptom levels.

**Results:**

Hypervigilance significantly predicted anxiety symptoms at follow-up (β = .250, p = .003), accounting for an additional 4.1% of the variance beyond baseline anxiety. In contrast, its predictive value for depressive symptoms was non-significant (β = .119, p = .080). These findings suggest that hypervigilance may serve as a specific marker for anxiety vulnerability.

**Conclusions:**

Self-reported hypervigilance demonstrates longitudinal specificity in predicting anxiety symptoms, but not depressive symptoms. These results support its potential utility in early identification and prevention strategies targeting anxiety disorders.

**Disclosure of Interest:**

C. Sorroche: None Declared, L. Prat-Torres: None Declared, P. Chavarría-Elizondo: None Declared, V. De la Peña-Arteaga: None Declared, I. Martínez-Zalacaín: None Declared, A. Juaneda-Seguí: None Declared, E. Vilajosana: None Declared, J. Raduà Paid Instructor of: Dr. Raduà reports receiving continuing medical education honoraria from Inspira Networks for a machine learning course promoted by Adamed, outside the submitted work., C. Soriano-Mas: None Declared, M. À. Fullana: None Declared

